# First case report of acute cholangitis secondary to *Cronobacter sakazakii*

**DOI:** 10.1186/s12879-021-06195-4

**Published:** 2021-05-27

**Authors:** Syeda Sahra, Abdullah Jahangir, Neville Mobarakai, Allison Glaser, Ahmad Jahangir, Muhammad Ans Sharif

**Affiliations:** 1 Staten Island University Hospital, 475-Seaview Avenue, Staten Island, NY 10305 USA; 2 King Edward Medical University, Lahore, 54000 Pakistan

**Keywords:** Cholangitis, Cholecystitis, *Cronobacter sakazakii*, Gallbladder infection

## Abstract

**Introduction:**

*Cronobacter sakazakii* is an opportunistic Gram-negative, rod-shaped bacterium which may be a causative agent of meningitis in premature infants and enterocolitis and bacteremia in neonates and adults. While there have been multiple cases of *C. sakazakii* infections, there have been no acute cholangitis cases reported in humans.

**Case presentation:**

An 81-year-old male with a past medical history of basal cell carcinoma, alcoholic liver cirrhosis, transjugular intrahepatic portosystemic shunt procedure, complicated by staphylococcus bacteremia, pituitary tumor, glaucoma, and hypothyroidism presented to the emergency room with the complaint of diffuse and generalized 10/10 abdominal pain of 1 day’s duration. There was a concern for pancreatitis, acute cholangitis, and possible cholecystitis, and the patient underwent a percutaneous cholecystostomy tube placement. Blood cultures from admission and biliary fluid cultures both grew *C. sakazakii*. The patient was treated with a carbapenem and clinically improved.

**Conclusions:**

The case study described a patient with multiple medical comorbidities that presented with *C. sakazakii* bacteremia and cholangitis. While this bacterium has been implicated in other infections, we believe this is the first time the bacteria is being documented to have caused acute cholangitis.

## Background

*C. sakazakii* is responsible for life-threatening infections, including meningitis and brain abscesses with potentially permanent neurological sequelae for the neonatal population. The CC4 variant of *Cronobacter* has been associated with necrotizing enterocolitis as well. Urinary tract infections, septicemia, and lung and wound infections are reported in adults, particularly in those with underlying immunocompromising comorbidities. We present the first case of acute cholangitis in an elderly male where *C. sakazakii* was reported on the PCR assay of cultures from blood and biliary fluid. This is the first case of *C. sakazakii* in the medical literature where it caused cholangitis in an adult patient to the best of our knowledge. The patient responded well to therapy with a carbapenem. Infection with *C. sakazakii* should be considered in cholangitis in the elderly population with comorbidities not responsive to cephalosporins.

## Case presentation

An 81-year-old male came to E.R. with the complaint of abdominal pain of 1 day’s duration. His past medical records were significant for basal cell carcinoma, alcoholic liver cirrhosis with a history of an intrahepatic portosystemic shunt procedure, complicated by staphylococcus bacteremia, pituitary tumor, glaucoma, and hypothyroidism. The abdominal pain had been constant, non-radiating, diffuse, burning in nature, 10/10 in intensity, and associated with chills. He denied any associated fevers, cough, nausea, vomiting, diarrhea, chest pain, headaches, shortness of breath, recent travels, or sick contacts.

On physical examination, his temperature was 98 °F, pulse 104 bpm, blood pressure 133/74 mmHg, respiratory rate 18 beats per minute, O2 saturation 99%. An abdominal exam revealed an obese body habitus, distention, and periumbilical tenderness with a negative Murphy’s sign.

**Fig. 1:**
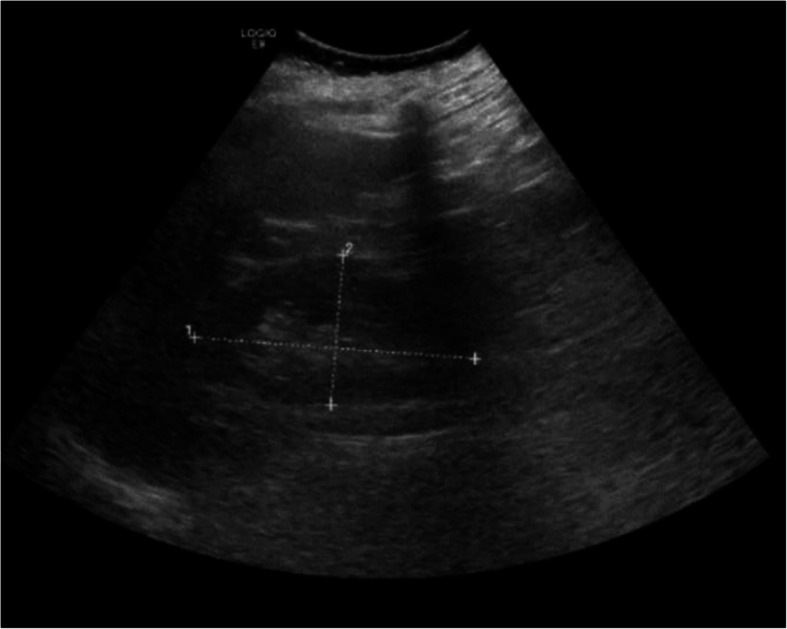
Ultrasound on arrival showing biliary sludge and cholelithiasis without any radiographic evidence of acute cholecystitis

**Fig. 2:**
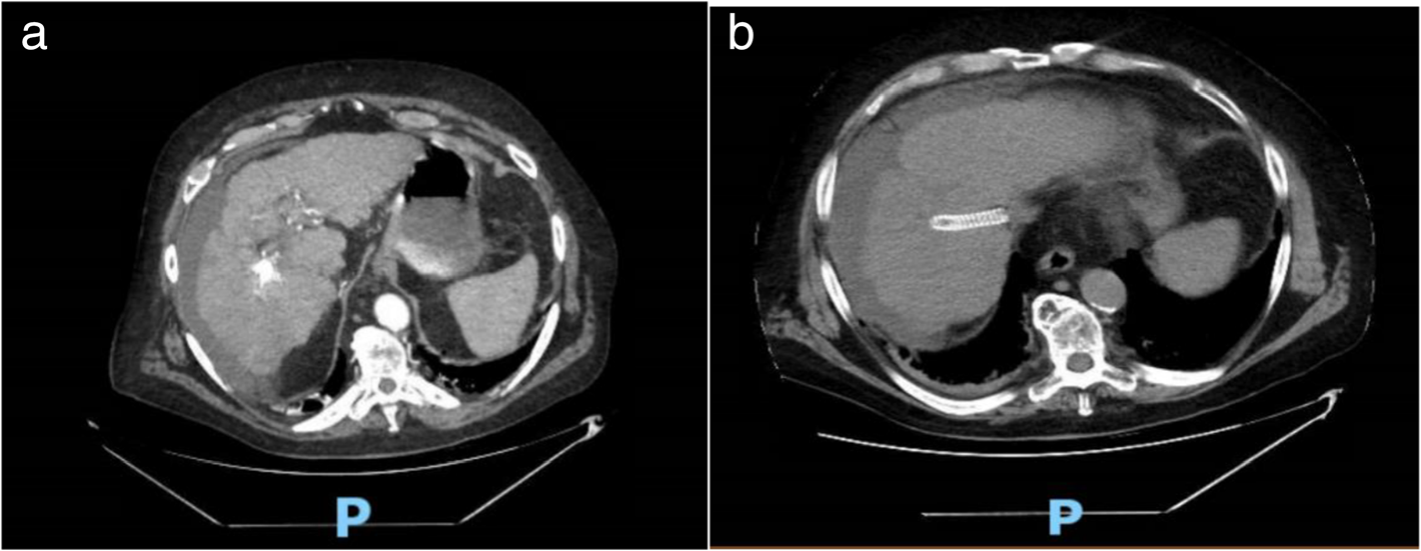
**a** & **b** C.T. scan abdomen showing nodular contour of liver, perihepatic ascites and TIPS from prior intervention

Initial blood work showed normal leukocyte count white blood cells (WBC) 4.5 K/uL, Creatinine 1.2 mg/dL, elevated aspartate aminotransferase (A.S.T.) 62 U/L, elevated Lipase 451 u/L, elevated Lactate 8.8 mmol/L, and normal serum levels of alanine aminotransferase (A.L.T.) 28 U/L, Alkaline Phosphatase 99 U/L, and total serum bilirubin 1.1 mg/dL. The sonogram and C.T. scan of the abdomen revealed perihepatic ascites, nodular liver contour, cholelithiasis, and gallbladder sludge with mild gallbladder wall thickening up to 4 mm without any radiographic evidence of acute cholecystitis (Figs. 1 and 2).

The patient was not a surgical candidate due to his advanced age and multiple comorbidities, and a CT-guided percutaneous cholecystotomy was performed for drainage of biliary fluid by Interventional Radiology. The biliary fluid was sent for culture, and cefepime and metronidazole were started empirically.

On the second day of hospitalization, the periumbilical pain improved, but right upper quadrant tenderness increased, and Murphy’s sign was elicited. The patient concurrently developed fever, leukocytosis (16.5 k/uL) with predominant neutrophilia (81%), and worsening acute kidney injury. Blood specimens drawn on admission were positive for gram-negative rods, which speciated to *C. sakazakii* on PCR studies. The PCR assay was performed using BioFire FilmArray.

**Fig. 3:**
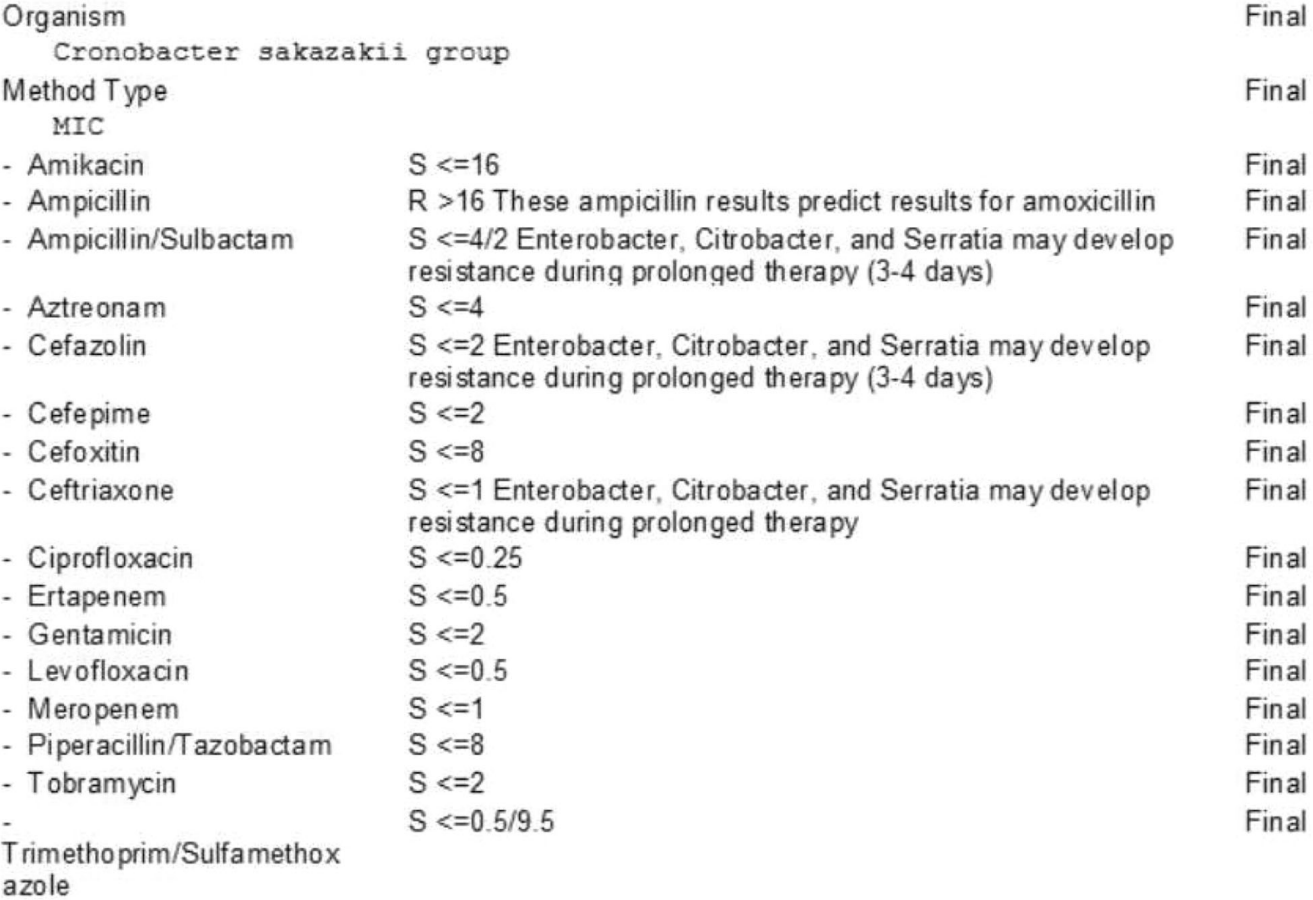
Antibiotic sensitivities and susceptibilities for blood cultures growing *Cronobacter sakazakii*

**Fig. 4:**
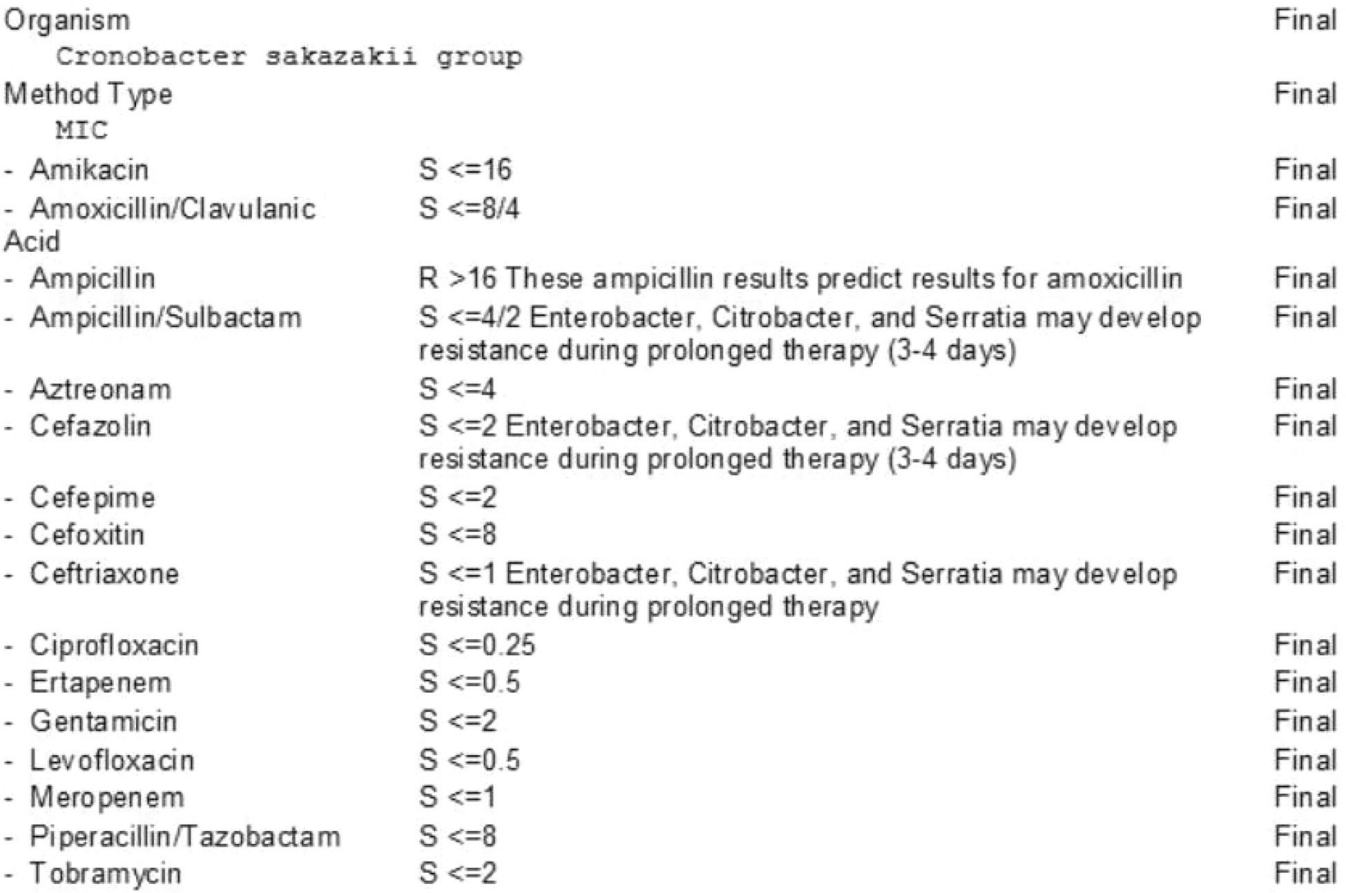
Antibiotic sensitivities and susceptibilities for cultures obtained from biliary fluid growing *Cronobacter sakazakii*

Body fluid samples underwent protein extraction, priming, and drying before matrix-assisted laser desorption/ionization-time of flight (MALDI-TOF) mass spectrometry was applied to the resultant intact cells and extracts from cells. Identification targets other than *C. sakazakii* were *Enterococcus* including vancomycin-resistant *Enterococcus, L. monocytogenes,* coagulase-negative *Staphylococcus, S. aureus, S. agalactiae* (Group B)*, S. pneumoniae, S. pyogenes* (Group A)*, A. baumannii, E. cloacae, E. coli, K. oxytoca, K. pneumoniae, Proteus sp., S. marcescens, H. influenzae, N. meningitidis, P. aeruginosa, C. albicans, C. glabrata, C. krusei, C. parapsilosis, C. tropicalis* and the *K. pneumoniae* carbapenemase (KPC) resistant gene. Microflex L.T. and MADLI Biotyper by Bruker Daltonics, Germany was employed in our institutional laboratory for mass spectrometry measurements and microorganism identification. The same organism was found in the biliary drain culture, confirming acute cholangitis due to *C. sakazakii* complicated by bacteremia. Susceptibilities of *C. sakazakii* were obtained against 17 antibiotics (Figs. 3 and 4).

Antibiotic coverage was consequently expanded to IV meropenem. His clinical picture and blood work improved exponentially with a downtrend of leukocytes, lactate, lipase, bilirubin, and liver enzymes (Table 1). He was discharged home on hospital day seven on oral ciprofloxacin and metronidazole for 10 days and was reported doing well on telephonic follow-up. The patient was later seen in outpatient clinics (Surgery and Infectious diseases) 2 weeks later. He was asymptomatic without any residual disease, and the biliary drain was removed successfully. The patient underwent an elective cholecystectomy a few weeks later with no complications.

**Table 1:** Trend of pertinent blood work through the hospitalization

Blood work	Day 1	Day 2	Day 3	Day 4	Discharge blood work
**WBC count (k/uL)**	4.46	16.59	13.62	10.59	5.11
**Hemoglobin (g/dL)**	13.5	12.1	11.1	10.9	11.2
**Creatinine (mg/dL)**	1.2	1.8	1.3	1.0	0.9
**BUN (mg/dL)**	16	38	36	29	14
**Bilirubin Total, serum (mg/dL)**	1.1	2.5	2.3	1.3	1.2
**AST (U/L)**	62	181	250	170	40
**ALT (U/L)**	28	49	81	67	31
**Lactate (mmol/L)**	8.8	7.2	5	4.3	1.5
**Lipase (U/L)**	451	44			

## Discussion and conclusions

*C. sakazakii*, previously called *Enterobacter sakazakii*, has been notorious since the 1980s for its fatal complications, including meningitis, hydrocephalus, and brain abscess in the neonatal population [1, 2]. *C. sakazakii* infections, including urinary tract infections, bacteremia, osteomyelitis, splenic abscess, and wound infections, have been reported in the adult population, particularly in those with immunocompromising conditions [3–7]. A clinical study on stroke patients associated it with aspiration pneumonia as higher oral *C. sakazakii* inoculums were reported in patient cohorts [8].

*C. sakazakii* has been reported in the human gastrointestinal tract, domestic and nosocomial settings, laboratory samples, and food items [9–13]. Bacterial contamination of infant formula milk was thought accountable for outbreaks in the neonatal population [14–19]. Studies have been done and are in the process of establishing the true etiology and source of *C. sakazakii*, which previously had been a diagnostic challenge leading to lesser reporting [20].

The case presented is the first case of cholangitis secondary to *C. sakazakii* in humans. There was no radiographic evidence of cholecystitis. The source of this Gram-negative rod invasion could be from the patient’s gastrointestinal flora. Basal cell carcinoma, liver cirrhosis, advanced age, and being on long-term steroid maintenance therapy could have contributed to immunosuppression and exponential growth of the opportunistic pathogen in the colon. Studies have reported *C. sakazakii* in pig gallbladder, hinting towards the gallbladder being a potential site of colonization and the risk of contracting *C. sakazakii* from consuming pork meat [21]. We could not rule out a dietary source in this patient. Sample contamination or infection with opportunistic gastrointestinal pathogens including *Klebsiella, Acinetobacter, and E. coli* were ruled out as *C. sakazakii* with similar antibiotic sensitivity was found on PCR studies for both blood biliary fluid. Besides, there was a dramatic response to optimum antibiotic treatment.

Carbapenems have been particularly efficient as *C. sakazakii* is resistant to ampicillin and the majority of cephalosporins [22]. Resistance to ampicillin was reported on antibiotic susceptibility testing for this organism as well. Ceftazidime, cephalothin, and cefotaxime were not added to the antibiotic sensitivity panel, which is a shortcoming in this case. The case raises the fact that *C. sakazakii* is a potential causative organism for gallbladder infections, particularly in elderly populations with underlying comorbidities. In conclusion, this case is significant in its clinical manifestation, causative pathogen, infection site, and treatment.

## Data Availability

The datasets used and/or analyzed during the current case reports are available from the corresponding author on reasonable request.

## Abbreviations

E.R.: Emergency room
MALDI-TOF MS: Matrix-assisted laser desorption ionization-time of flight mass spectrometry
